# Extracellular vesicles derived from endometrial epithelial cells deliver exogenous miR-92b-3p to affect the function of embryonic trophoblast cells via targeting TSC1 and DKK3

**DOI:** 10.1186/s12958-022-01023-z

**Published:** 2022-10-25

**Authors:** Renwu Hua, Qiaorui Liu, Weisi Lian, Ting ting Kang, Dengying Gao, Cheng Huang, Yueying Wang, Minggang Lei

**Affiliations:** 1 Key Laboratory of Agricultural Animal Genetics, Breeding and Reproduction of the Ministry of Education and Key Laboratory of Swine Genetics and Breeding of the Ministry of Agriculture, College of Animal Science and Technology, Huazhong Agricultural University, Wuhan, 430000 China; 2 Shenzhen Key Laboratory of Fertility Regulation, Center of Assisted Reproduction and Embryology, The University of Hong Kong-Shenzhen Hospital, Shenzhen, 518053 China; 3 Center for Energy Metabolism and Reproduction, Shenzhen Institute of Advanced Technology,Chinese Academy of Sciences, Shenzhen, 518055 China; 4 Scientific Research Center, The Seventh Affiliated Hospital, Sun Yat-Sen University, Shenzhen, 518000 China; 5 Department of Reproductive Medicine, Jining No.1 People’s Hospital, Jining, 272000 China

**Keywords:** Extracellular Vesicles, miR-92b-3p, *TSC1*, *DKK3*, Embryo implantation

## Abstract

**Background:**

Extracellular vesicles (EVs) could mediate embryo-maternal communication to affect embryo implantation by delivering biology information, including microRNA (miRNA), protein, lipid. Our previous research shows that miR-92b-3p was differentially expressed in EVs of uterine flushing fluids during the embryo implantation period. However, the role of miR-92b-3p from EVs in embryo implantation remains elusive.

**Materials and methods:**

EVs were isolated from porcine endometrial epithelial cells (EECs) by ultracentrifugation. MiR-92b-3p mimics and EVs were used to regulate the expression of miR-92b-3p in porcine trophoblast cells (PTr2 cells). Cell proliferation, migration and adhesion analyses were used to observe the phenotype. RT-qPCR, western blot and dual-luciferase reporter assay were used to assess the targets of miR-92b-3p.

**Results:**

In this study, EVs derived from porcine EECs were identified and could be taken up by PTr2 cells. We found that the EVs derived from EECs transfected with miR-92b-3p mimic (EVs-miR-92b-3p) significantly promoted the proliferation, migration and adhesion of PTr2 cells. We verified that *Tuberous sclerosis complex subunit* (*TSC1*) and *Dickkopf 3* (*DKK3*) were the target genes of miR-92b-3p. Moreover, our study showed that miR-92b-3p plays a vital role in PTr2 cells via targeting *TSC1* and *DKK3.* Furthermore, the 3'UTR vectors of TSC1 and DKK3 can rescue the effect of miR-92b-3p on PTr2 cells.

**Conclusions:**

Taken together, this study reveals a novel mechanism that EVs derived from porcine EECs treated with miR-92b-3p crosstalk with trophoblasts by targeting *TSC1* and *DKK3*, leading to an enhanced ability for implantation.

**Supplementary Information:**

The online version contains supplementary material available at 10.1186/s12958-022-01023-z.

## Background

Approximately 30–40% of pre-natal losses occur in the pigs, and it is mainly caused by the failure of embryo implantation [1, 2]. During the embryo implantation, the blastocyst gradually establishes physiological and structural connections with the maternal endometrium tissue and form the placenta that connects the growing embryo to the maternal uterine circulation [3]. The synchronization of maternal–fetal communication exerts a significant impact on the success of embryo implantation [4]. Notably, the extracellular vesicles (EVs) of uterine fluids could mediate intrauterine maternal–fetal cross-talk during the embryo implantation period [5, 6]. However, literature on the mechanism of action of EVs of uterine fluids in the communication between embryos and maternal endometrium is scant.

EVs, including exosomes, apoptotic bodies and microvesicles, are considered to be effective carriers for intercellular communication in both prokaryotes and eukaryotes [7–9]. Previous studies have reported that EVs were found in uterine cavity fluids and were widely recognized as an important factor that influences embryo implantation in humans, mice, cattle, sheep and pigs [10–14]. These vesicles can play a role by transmitting biological information to the recipient cells to affect the function of the receptor cells [15]. EVs can carry different types of biomolecules (such as miRNA, proteins, lipids, and DNA) to mediate signal transduction, and provide a unique package for the transmission of biological information to protect them [16–18]. The EVs cargos derived from endometrial epithelial cells (EECs), especially miRNAs (such as miR-100-5P and miR-30d), exert a significant impact on the proliferation, adhesion and migration of trophoblast cells [14, 19]. Our previous study revealed that miR-92b-3p was differentially expressed in EVs from porcine uterine flushing fluids during the embryo implantation period [20]. The miR-92b-3p derived from EVs also has strong effects on cell proliferation and migration [21] However, the role and the regulatory mechanism of EVs miR-92b-3p in embryo implantation are not well understood.

In this study, we found that miR-92b-3p was differentially expressed not only in EVs of uterine flushing fluids but also in endometrial tissue during porcine embryo implantation. Furthermore, we aimed to investigate the role of and mechanisms by which the miR-92b-3p in EVs derived from EECs act as regulators of porcine trophoblast cells. Our study provides important insights into the intrauterine endometrial-embryonic communication during porcine embryo implantation.

## Methods

### Tissues collection

Nine purebred Yorkshire gilts with similar weights (120 ± 10 kg), ages (8 months) and genetic background were selected in this study. Gilts were artificially inseminated using extended semen from one boar (the breeding pig farm of Huazhong Agricultural University) at the onset of estrus (day 0) and again 12 h later. Uteri and embryos were obtained from pigs slaughtered on days 10, 13 and 18, and uterine flushing fluids were collected by flushing three times with 30 mL phosphate buffer saline (PBS; pH 7.4, 02–024-1ACS, Biological Industries, Israel). The pregnancy was confirmed by the size and morphology of conceptuses as follows: day 10 (spherical conceptuses with a diameter of 2–8 mm), day 13 (filamentous forms of conceptuses) and day 18 (trophoblast tissue and embryos with evident vascularization). The endometrial tissues from pregnancy gilts were used for subsequent analyses.

### EVs isolation and preparation

The cell culture medium was clarified by centrifugation (2000 g for 30 min at 4 °C) to remove whole and dead cells. The supernatant was subjected to a second centrifugation at 10,000 g for 30 min to remove cells debris. The supernatants were filtered through a 0.22 μm filter (MILLEX-GP, USA) to remove bacteria and impurities. Then, these samples were ultracentrifuged at 130,000 g for 2 h at 4 °C (Beckman Optima XE-90, SW32 Ti rotor, Beckman Coulter. USA). EVs were re-suspended in 200 μL PBS (catalog number: 02–024-1ACS, Biological Industries) after washing with PBS (130,000 g for 2 h). EVs were stored at –80 °C until the RNA was isolated. Protein concentration in the final EV pellet was measured by BCA (Pierce, Thermo Fisher Scientific) according to the manufacturer’s instructions.

### NTA

Isolated EVs were diluted with PBS at the ratio of 1:200 and added into the chamber. The size distribution of isolated EVs was analyzed by NTA with Zetasizer Nano ZS (Malvern Panalytical).

### TEM

The morphology of isolated EVs was visualized with high-resolution transmission electron microscope (Hitachi HT7700) based on a previous method [22]. In brief, the resuspended EVs were placed on a carbon-coated copper grid, and then subjected to 2% phosphotungstic acid (DZ0035, Leagene, Beijing, China) staining for 2 min. The excess liquid was blotted off by filter paper, and grids were allowed to dry overnight.

### Species conservation analysis of miRNAs

The sequences of miRNAs in different species were obtained through miRbase (https://www.mirbase.org/). The conservation analysis was performed by comparing mature sequences of miRNAs from different species.

### EVs-free cell culture medium preparation

The serum following 1:4 dilution was ultra-centrifuged with an ultracentrifuge (1,300,000 g, 18 h) to remove EVs [23]. After centrifugation, the supernatant was used to prepare the complete medium.

### Cell culture

Six additional non-pregnant gilts (day12 of estrous cycle) at a similar age (8 months) and weight (120 ± 10 kg) were slaughtered for in vitro culture of endometrial epithelial cells. Isolation and culture of porcine primary endometrial epithelial cells (EECs) referred to previous research [24]. The endometrium was separated and shredded with a sterile scissor. After washing twice with PBS, the tissue pieces were incubated at 37 °C for 2.5 h with collagenase I (Gibco, NY, USA) and shaken vigorously every half hour. Undigested tissue pieces were removed by screen filtration. Then, the filtrate was centrifuged at 500 g for 10 min to remove supernatant (fraction rich of endometrial stromal cells). The pellet (epithelial‐rich fraction) was resuspended twice in PBS and recentrifuged (500 g, 10 min) twice. The resultant pellets containing EECs were suspended in Dulbecco’s modified Eagle’s medium/F-12 (DMEM/F12; 1:1) medium (Gibco) supplemented with 10% fetal bovine serum (Gibco) and 1% penicillin–streptomycin (Gibco) and cultured in 37 °C and 5% CO_2_ incubator. Endometrial stromal cells were further removed by 0.25% trypsin without edetic acid disodium salt (EDTA) after 2–4 d. The epithelial cells (purity > 95%) characterized for epithelium-specific cytokeratin positive were then trypsinized with 0.25% trypsin–EDTA and placed on Cell Culture Flask (Corning, NY, USA) for subsequent experiments [24].

Porcine trophectoderm cells (PTr2 cells) were kindly provided by Mr. Jiang zongyong, Guangdong Academy of Agricultural Sciences. PTr2 cells were established from dispersed cell culture of day 12 filamentous conceptus obtained from pigs. These cells have been previously characterized for SN1/38 (porcine trophectoderm-specific monoclonal antibody) positive, cytokeratin 7 positive, vimentin negative, express fibronectin and many of the integrin subunits present in porcine trophectoderm in *vivo*. Detailed methods have been published [25]. These cells were cultured in DMEM-F12 (Gibco) supplemented with insulin (0.1 Units/mL; Sigma-Aldrich, German), glutamine (2 mM, Sigma-Aldrich), 1% penicillin–streptomycin and 5% fetal bovine serum (Gibco).

### The uptake of CM-Dil labelled EVs

To monitor EVs trafficking, EVs were labeled with CM-Dil fluorescent dye using the Celltracker CM-Dil kit (Yeasen). Briefly, EVs were mixed with 1 μM CM-Dil, and the EVs–dye suspension was incubated for 30 min in the 37 °C incubator. After CM-Dil staining, the EVs were washed in 35 ml PBS and collected by ultracentrifugation (130,000 × g for 70 min) at 4ºC. Finally, CM-Dil labeled EVs were resuspended in PBS and added to PTr2 cells. After 6 h or 12 h, the PTr2 cells were dyed with AbFluor™ 488-Phalloidin Kit (Abbkine) according to the manufacturer’s instructions. These PTr2 cells were observed via laser scanning confocal microscope (Zeiss LSM 800).

### Co-culture assay

After transfection with FAM-miR-92b-3p mimic, EECs were co-cultured with PTr2 cells at a ratio of 1:1 using a trans-well plate (0.4 mm polycarbonate filter, Corning) for 24 h, with PTr2 cells placed in the lower chamber and EECs placed in the upper chamber. After washing with PBS twice, the appearance of FAM green fluorescence on PTr2 cells was examined.

### RNA extraction and real time quantitative PCR (RT-qPCR)

The RNA was extracted from cells using Trizol (Invitrogen) as recommended by the manufacturer and the concentration and quality were measured by the NanoDrop 2000 (ThermoFisher, Waltham, USA). The complementary DNA (cDNA) was synthesized with a reverse transcription kit (Takara, Tokyo, Japan). Then, the Mix (Toyobo, Japan) and specific primers for every gene (Table S1) were used to perform RT-qPCR on a Real-time System (Roche, Basel, Switzerland). The expression levels of miR-92b-3p and genes were normalized with U6 and RPS20 to obtain the relative expression using the 2^–ΔΔCt^ method, respectively.

### Transfection

All RNA oligonucleotides were designed and synthesized by GenePharm (Shanghai, China) and are shown in Table S2. EECs or PTr2 cells were transfected with 100 nM miRNA agomirs in 6-well plates with Lipofectamine 2000 (Invitrogen, Waltham, USA) according to the manufacturer’s instructions.

### Cell proliferation assay

The Cell Counting kit-8 (CCK-8, Dojindo, Shanghai, China) was used to measure cell proliferation after transfection 48 h following the manufacturer's instructions. The optical density (OD) at 450 nm of each well plate was determined using a microplate reader (Bio-Rad, CA, USA).

### Cell migration assay

Cell migration was assessed by Transwell assay that had 12 mm polycarbonate membranes of 8.0 μm pore size (Corning, NY, USA). The cells were re-suspended with serum-free medium as a single-cell solution at 4 h post-transfection. About 2 × 10^5^ cells were seeded on the upper champers, and complete Medium with10% FBS was added to the lower chamber as a chemoattractant. After 24 h of incubation at 37 °C with 5% CO2, cells which migrated to the lower chamber were fixed with 4% paraformaldehyde for 5 min, stained with 0.1% crystal violet for 5 min, rinsed third in PBS and subjected to OLYMPUS DP80 microscope (Tokyo, Japan). The number of migration cells was obtained by counting five fields per membrane and derived from three independent experiments.

### Cell adhesion

Cell adhesion was detected with reference to the previous method [26]. Firstly, matrigel solution (Corning, USA) was 1:5 diluted by serum-free medium, and then they were added into 96-well plate (20 μl/well) for 1 h at 37 °C. Secondly, the density of cells was adjusted to 1 × 10^5^/ml using serum-free medium, and then they were added into the same 96-well plate (100 μl/well), which was incubated at 37 °C with 5% CO2 for 1 h. After that, the medium was removed, and the non-adherent cells were washed away by PBS, followed by adding serum-free medium into 96-well plate (200 μl/well). Thirdly, CKK-8 solution was added to the 96-well plate (10 μl/well), incubating for 4 h. After that, the OD value of each well was detected by a microplate reader (λ = 450 nm). Finally, the rate of adherent cells (%) was estimated using the following formula: the rate of adherent cells (%) = (Experimental group – Negative control group) / (Negative control group) *100%

### Western blot

The exosomal and cellular proteins were extracted by DNA/RNA/protein Isolation Kit (catalog number: R6734-02, OMEGA). The sample was denatured by heating, separated by SDS-PAGE and transferred to a PVDF (polyvinylidene fluoride) membrane. Next, the membranes were blocked with 5% skimmed milk powder and separately probed with rabbit anti TSG101 (catalog number: GB11618, servicebio), rabbit anti HSP70 (catalog number: GB11241, servicebio), rabbit anti TSC1 (catalog number: GB11882, servicebio), rabbit anti calnexin (catalog number: 10427–2-AP, Proteintech), rabbit anti GAPDH (catalog number: GB11002, servicebio), rabbit anti β-actin (catalog number: GB11001, servicebio) and mouse anti DKK3(catalog number: 66758–1-Ig, Proteintech) overnight at 4 °C with a final dilution 1:1000 (v/v) in 5% milk. After three times of washing, the membranes were incubated with goat anti-rabbit secondary antibodies (GB23303, Sevicebio, Wuhan, China) or goat anti-mouse secondary antibodies (GB23301, Sevicebio, Wuhan, China) with 1:2000 dilution (v/v) at 37 °C for 1.5 h. The images of membranes treated with ECL (enhance chemiluminescence) were captured by Western Blotting Detection System (Tiangen, Beijing, China).

### Plasmid construct and dual-luciferase reporter assay

The miR-92b-3p target genes were predicted using a miRBase (http://www.mirbase.org/), TargetScan (http://www.targetscan.org/) and miRDB (http://www.mirdb.org/miRDB/). To construct reporters for luciferase assays, the fragment contained the binding sites of miR-92b-3p on the 3' UTR of TSC1 or DKK3 was cloned into the Pmir-GLO Vector (Promega, Madison, USA). The mutant of ssc-miR-92b-3p binding sites on the 3' UTR was generated using mutagenic primers (Table S3) to construct mutant vector (Pmir-GLO-TSC1-Mut or PmirGLO-DKK3-Mut). MiR-92b-3p and the dual-luciferase reporter vectors (PmirGLO-TSC1-WT, PmirGLO-TSC1-Mut, PmirGLO-DKK3-WT or PmirGLO-DKK3-Mut) were co-transfected into PTr2 cells. Treated cells were collected at 24 h post-transfection and the luciferase activity was detected with PerkinElmer 2030 Multilabel Reader (Promega, MDN, USA).

### Intrauterine injection of mice

It is difficult to perform in vivo experiments in pigs, so we used a murine implantation model based on other studies focusing on porcine or human implantation [27–29]. The date of finding the vaginal suppository after mating was designated as the first day. The intrauterine injection surgery under general anesthesia was performed on the eight mice on day 3 of pregnancy in the evening according to previous studies [28, 29]. 5 μL of 10 μM antagomiR-92b-3p and inhibitor negative control (NC) were injected into the left and right uterine horns, respectively. Then the wounds were sutured and the mice were put under a 37 °C warmer till awakening from the anesthesia. To reduce the pain of the mice, temgesic was injected into the mice at 12, 24, and 48 h after surgery. On day 7, five mice were killed and their uteri were isolated to record the number of implanted embryos.

### Statistical analysis

Data were expressed as means ± standard deviation (SD) derived from at least three independent experiments. Student's t-test (one tailed) was used to perform statistical analysis. *P* value < 0.05 was considered as statistically significant. * *P* < 0.05; ** *P* < 0.01.

## Results

### Characterization of extracellular vesicles derived from EECs

**Fig. 1:**
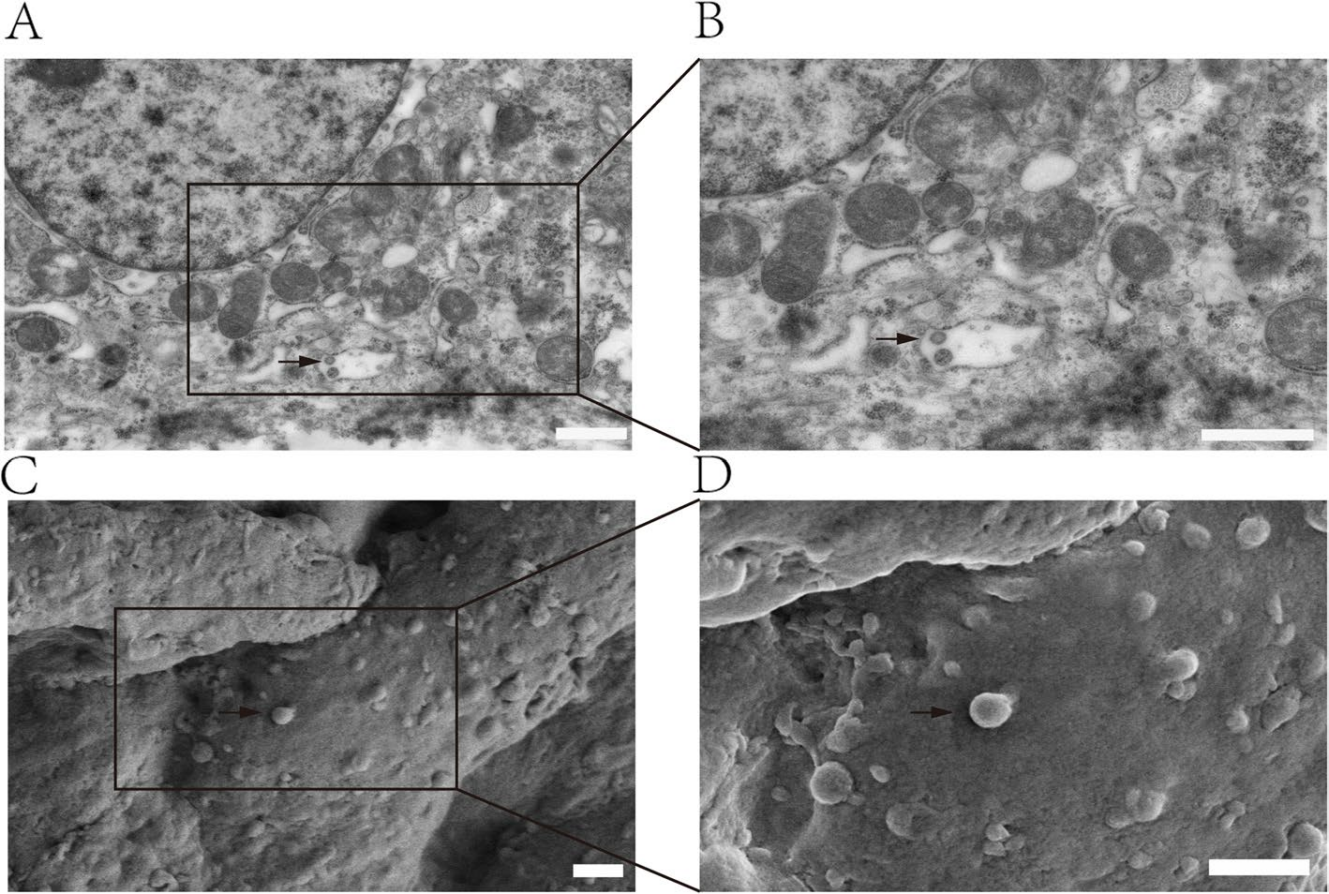
Observation of EVs in the uterus during porcine embryo implantation. **A B** Observation of the endometrium embryo implantation period with transmission electron microscope, scale bars, 1 μm. C **D** Observation of the embryos during embryo implantation period with scanning electron microscopy, scale bars, 1 μm

In order to identify the presence of EVs in uterine lumen during embryo implantation, we observed the presence of multivesicular bodies (MVBs) enclosing multiple cup-shaped vesicles in endometrial luminal epithelial cells on day 18 of pregnancy through transmission electron microscopy (Fig. 1 A, B). Scanning electron micrographs demonstrated that there are typical rounded vesicles (similar to EVs) attached to the surface of the embryo (Fig. 1 C, D).

**Fig. 2:**
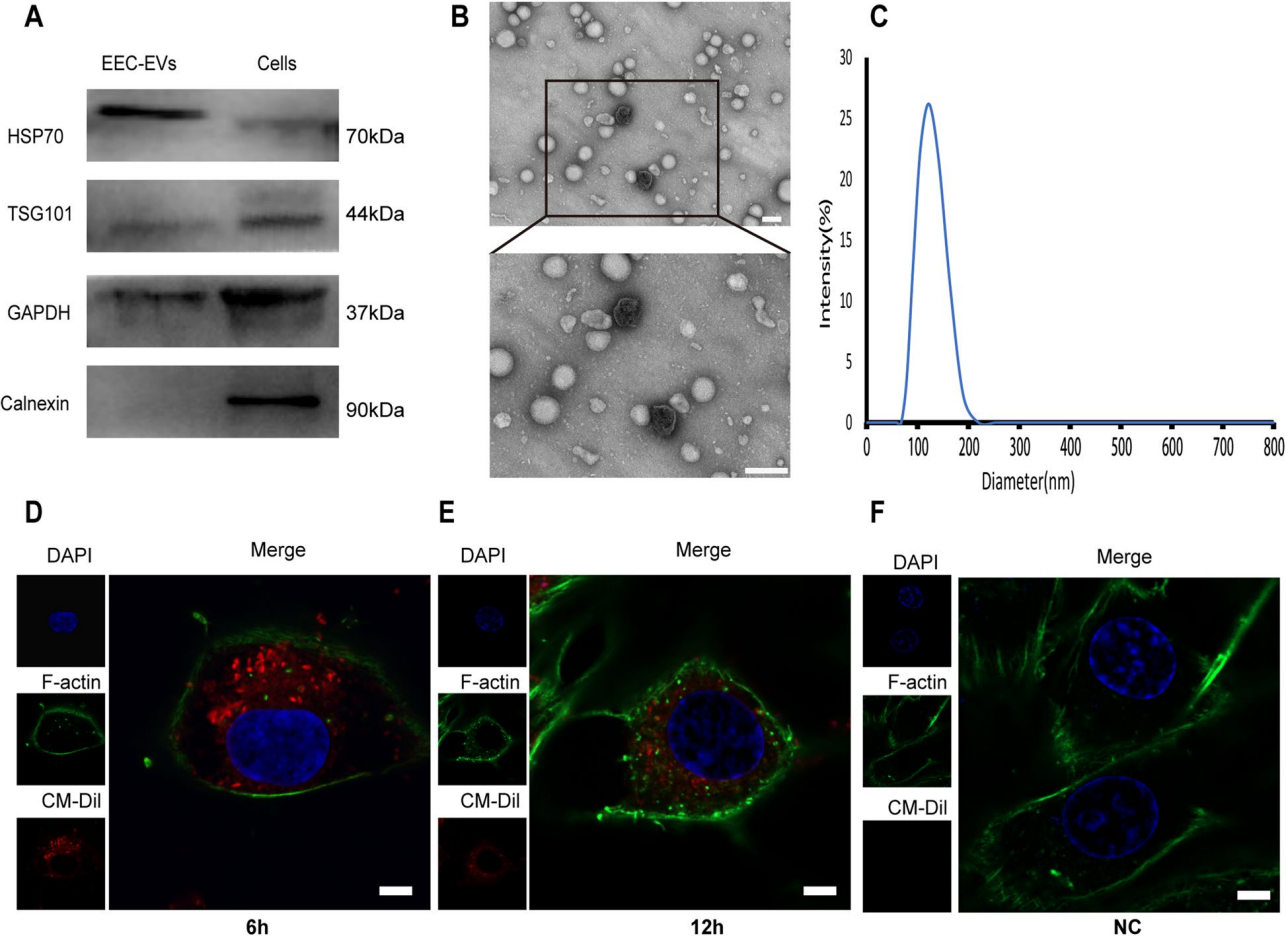
Characterization of the EVs derived from EECs. **A** Western Blotting detected HSP70, TSG101 and GAPDH in the EECs-EVs fraction as well as cellular fraction derived from PTr2 cells. Calnexin (negative control) was only detected in cell lysates of PTr2 cells. **B** Transmission electron microscopy analysis of EECs-EVs, Scale bar = 200 nm. **C** Particle size of the EVs from EECs-EVs was measured by Nanoparticle tracking analysis. **D** EECs-EVs (20 μ g/mL) labeled with CM-Dil were added to PTr2 cells culture medium for 6 h and 12 h. scale bars = 5 μm. NC is the supernatant of the stained EVs after ultra-isolation

A recent study has indicated that EVs in uterine lumen are mainly derived from EECs in pigs [30]. EVs derived from primary EECs were obtained from the conditional medium by filtration and ultracentrifugation. The results western blot revealed that the EVs fraction was positive for EVs-related protein markers (TSG101, HSP70 and GAPDH) and negative for calnexin (Fig. 2A). Transmission electron microscopy (Fig. 2B) and nanoparticle tracking analysis (Fig. 2C) revealed that the EVs were cup-shaped particles with a diameter of 80–190 nm. Then, we tested whether the EVs derived from EECs can be taken up by PTr2 cells. These EVs were labeled with CM-Dil and added into the culture medium of PTr2 cells. After 6 h or 12 h, red fluorescent dots were observed in PTr2 cells, indicating that the EVs were taken up by these cells (Fig. 2D, E).

### The expression profiles of miR-92b-3p

**Fig. 3:**
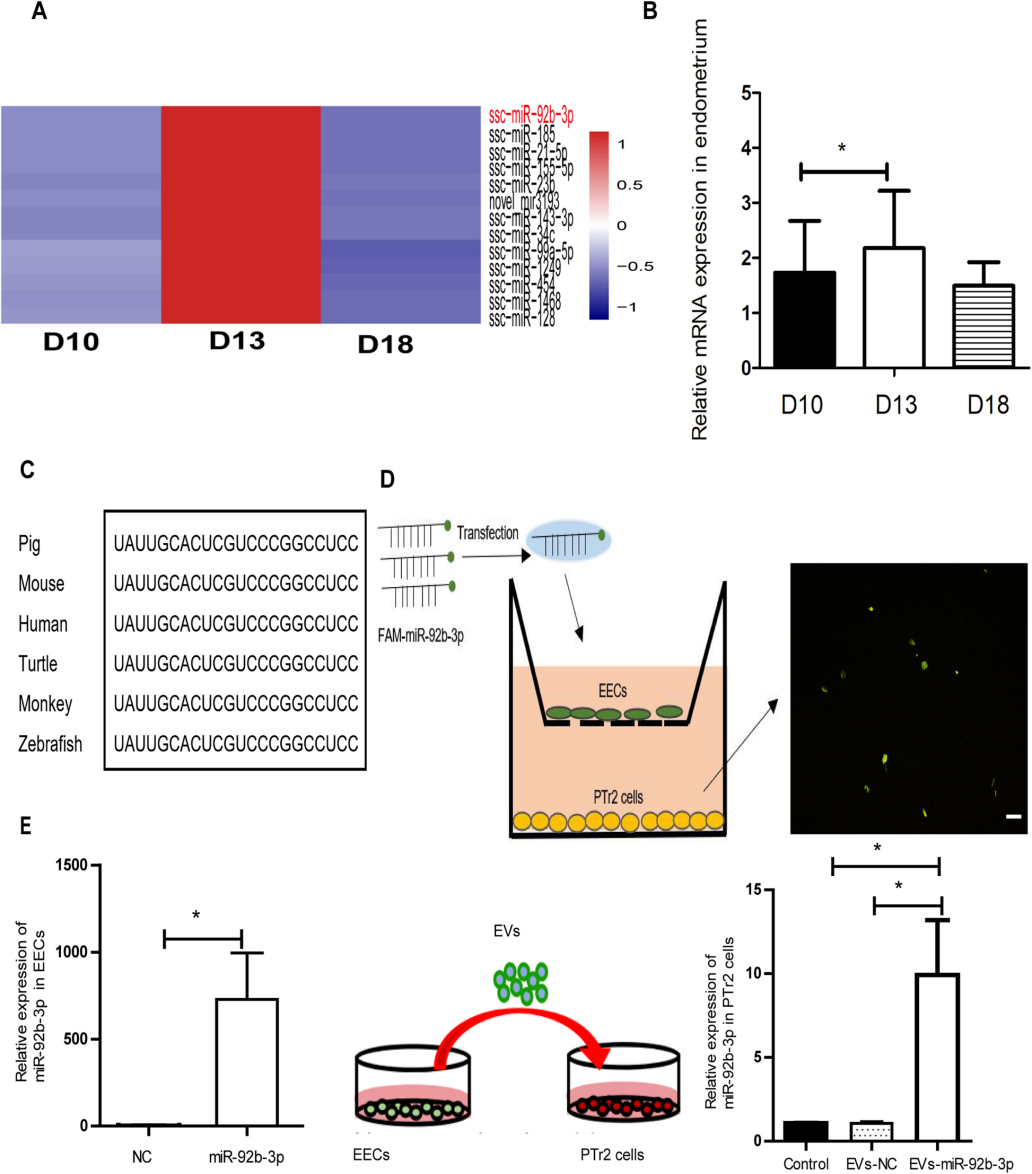
The expression profiles of miR-92b-3p. **A** A heatmap depicting the expression level of 13 miRNA in EVs from uterine flushing fluids on D10, D13 and D18. **B** The expression level of miR-92b-3p in porcine endometrium on D10, D13 and D18. **C** The conservation of miR-92b-3p from 6 different species. **D** The transmission of fluorescent miR-92b-3p in the transwell chamber. The EECs transfected with fluorescent miR-92b-3p were placed in the upper chamber, and the PTr2 cells were placed in the upper chamber. Scale bar = 50 μm. **E** After treating PTr2 cells with miR-92b-3p derived from EECs extracellular vesicles for 48 h, the expression of miR-92-3p increased significantly. Data are mean ± s.d., derived from three independent experiments. * *P* < 0.05, * *P < 0.01 (Student’s t-test)

Our previous research shows that the expression level of miR-92b-3p was higher in EVs from uterine flushing fluids on day13 of pregnancy than them on day 10 and day 18 of pregnancy (Fig. 3A) [24]. In the endometrial tissues of pigs, the expression level of miR-92b-3p was significantly upregulated from day 10 of pregnancy to day 13 of pregnancy (Fig. 3B). Noteworthily, the mature miR-92b-3p sequences are highly conserved in mammals including pigs, mice and humans (Fig. 3C). Then, we explored whether EECs can secrete extracellular miRNAs that are then delivered to PTr2 cells. EECs transfected with a fluorescent FAM-labeled miR-92b-3p mimic were co-cultured with PTr2 cells in a transwell plate (Fig. 3D). The presence of green fluorescent FAM dye in the PTr2 cells indicated that the FAM-miR-92b-3p mimic was transported from the EECs in the upper transwell to the recipient PTr2 cells placed in the lower well. Moreover, after the EVs derived from EECs transfected with miR-92b-3p mimic (EVs-miR-92b-3p) were added into the culture medium of PTr2 cells, a ninefold increase in miR-92b-3p expression was detected in the PTr2 cells (Fig. 3E).

### The role of EVs-miR-92b-3p in PTr2 cells

**Fig. 4:**
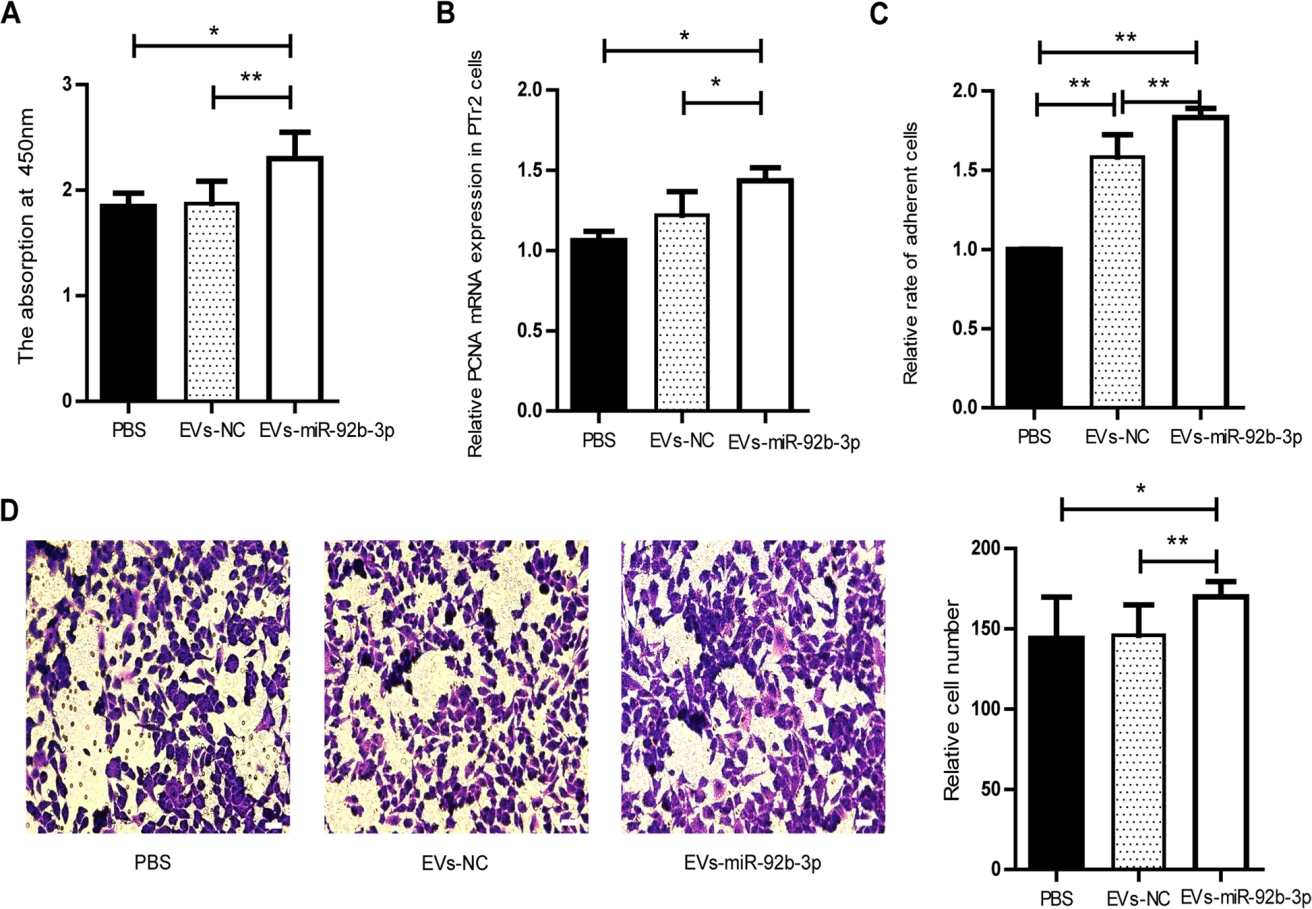
The effect of EVs-miR-92b-3p on PTr2 cells. **A** EVs-miR-92b-3p promoted the proliferation of PTr2 cells. **B** EVs-miR-92b-3p increased the mRNA expression level of PCNA in PTr2 cells. **C** The effect of EVs-miR-92b-3p on adhesion ability of PTr2 cells. **D** EVs-miR-92b-3p promoted the migration of PTr2 cells, scale = 20 μm. Data are mean ± s.d., derived from three independent experiments. * *P* < 0.05, * *P < 0.01

To explore the role of EVs-miR-92b-3p in PTr2 cells, the PTr2 cells were treated with EVs-miR-92b-3p. Our results showed that the treatment of EVs-miR-92b-3p significantly promoted proliferation and increased the mRNA expression level of PCNA in PTr2 cells (Fig. 4A, B). An adhesion experiment demonstrated that EVs-miR-92b-3p enhanced the adhesion ability of PTr2 cells (Fig. 4C). Notably, compared with PTr2 cells treated with PBS, the number of adhesion cells significantly increased in PTr2 cells treated with EVs-NC (EVs derived from EECs transfected with NC). In addition, EVs-miR-92b-3p remarkably elevated the migration of PTr2 cells (Fig. 4D). Altogether, EVs-miR-92b-3p promoted the proliferation, adhesion and migration of PTr2 cells.

**Fig. 5:**
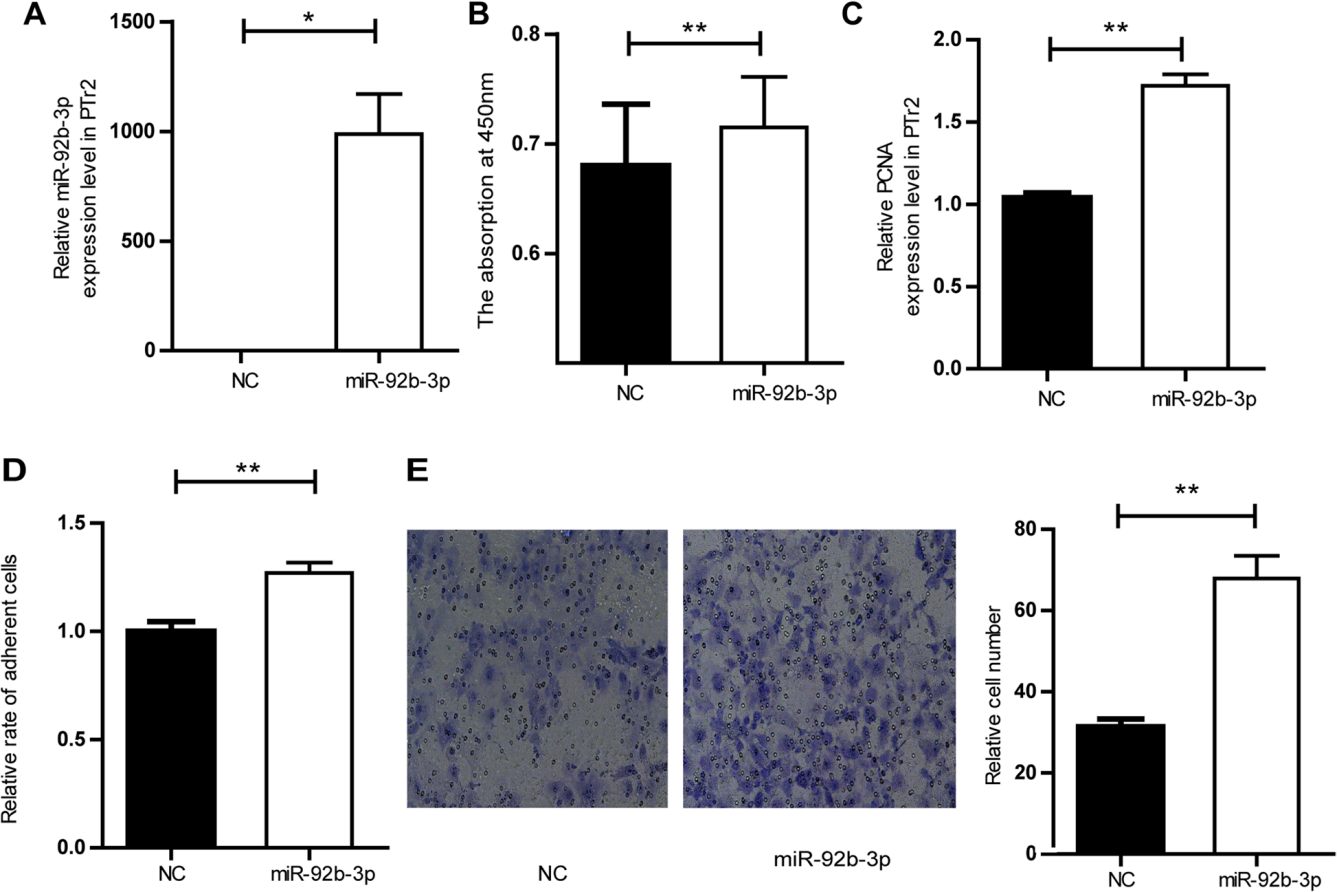
The effect of miR-92b-3p on PTr2 cells. **A** The miR-92b-3p mimic increased the expression level of miR-92b-3p in PTr2 cells. **B** The miR-92b-3p promoted the proliferation of PTr2 cells. **C** The miR-92b-3p mimic increased the mRNA expression level of PCNA in PTr2 cells. **D** The effect of miR-92b-3p on adhesion ability of PTr2 cells. **E** The miR-92b-3p promoted the migration of PTr2 cells, scale = 20 μm. Data are mean ± s.d., derived from three independent experiments. * *P* < 0.05, * *P < 0.01

**Fig. 6:**
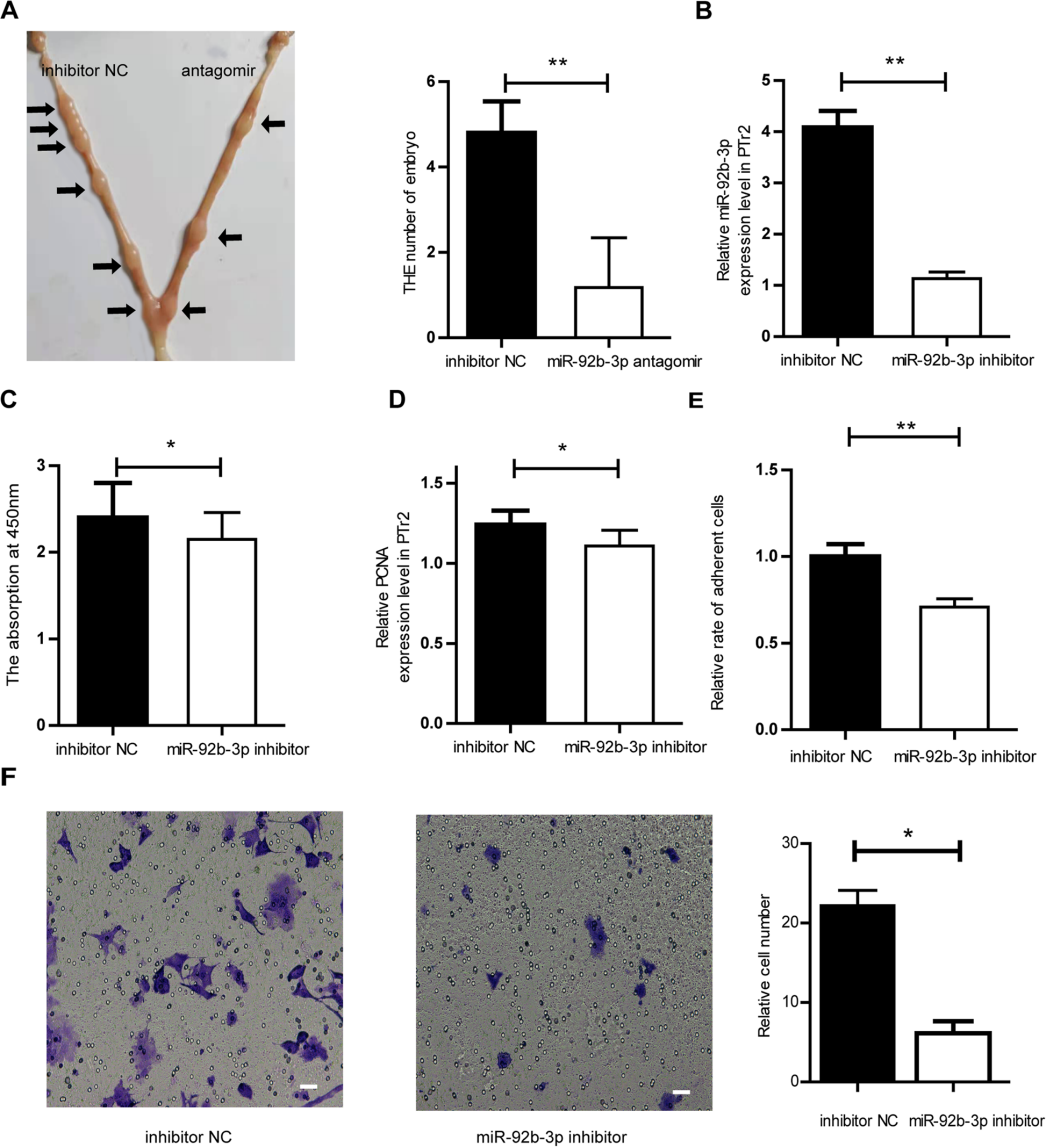
The effect of miR-92b-3p inhibitor on PTr2 cells. **A** Comparison of the number of implantation sites between inhibitor NC injected uterine horn (left) and miR-92b-3p antagomir injected uterine horn (right)(*n* = 5). **B** The miR-92b-3p inhibitor increased the expression level of miR-92b-3p in PTr2 cells. **C** The miR-92b-3p inhibitor inhibited the proliferation of PTr2 cells. **D** The miR-92b-3p inhibitor decreased the mRNA expression level of PCNA in PTr2 cells. **E** The effect of miR-92b-3p inhibitor on adhesion ability of PTr2 cells. **F** The miR-92b-3p inhibitor inhibited the migration of PTr2 cells, scale = 20 μm. Data are mean ± s.d., derived from three independent experiments. * *P* < 0.05, * *P < 0.01

To further estimate the role of miR-92b-3p in PTr2 cells, PTr2 cells were transfected with miR-92b-3p mimic or inhibitor. In PTr2 cells, the expression level of miR-92b-3p was remarkably up-regulated after the treatment of miR-92b-3p mimic (Fig. 5A). Our results indicated that miR-92b-3p enhanced the proliferation, adhesion and migration of PTr2 cells (Fig. 5 B, C, D, E). RT-qPCR experiments showed that miR-92b-3p increased the mRNA expression of IL1β, LIFR, c-Myc, OPN and inhibited the mRNA expression of Muc1 (Figure. 1S). We determined the effect of miR-92b-3p in embryo implantation by injecting miR-92b-3p antagomir or NC into the uterine horn on day 3 of pregnancy in mice. The number of implantation sites in the uterine horn injected with miR-92b-3p antagomir was less than in the uterine horn injected with NC on day 7 of pregnancy (Fig. 6A). After being transfected with miR-92b-3p inhibitor, the proliferation, adhesion and migration of PTr2 cells were hindered and the mRNA expression of PCNA was down-regulated (Fig. 6B, C, D, E, F).

### The miR-92b-3p directly targets 3’UTR of DKK3 and TSC1

**Fig. 7:**
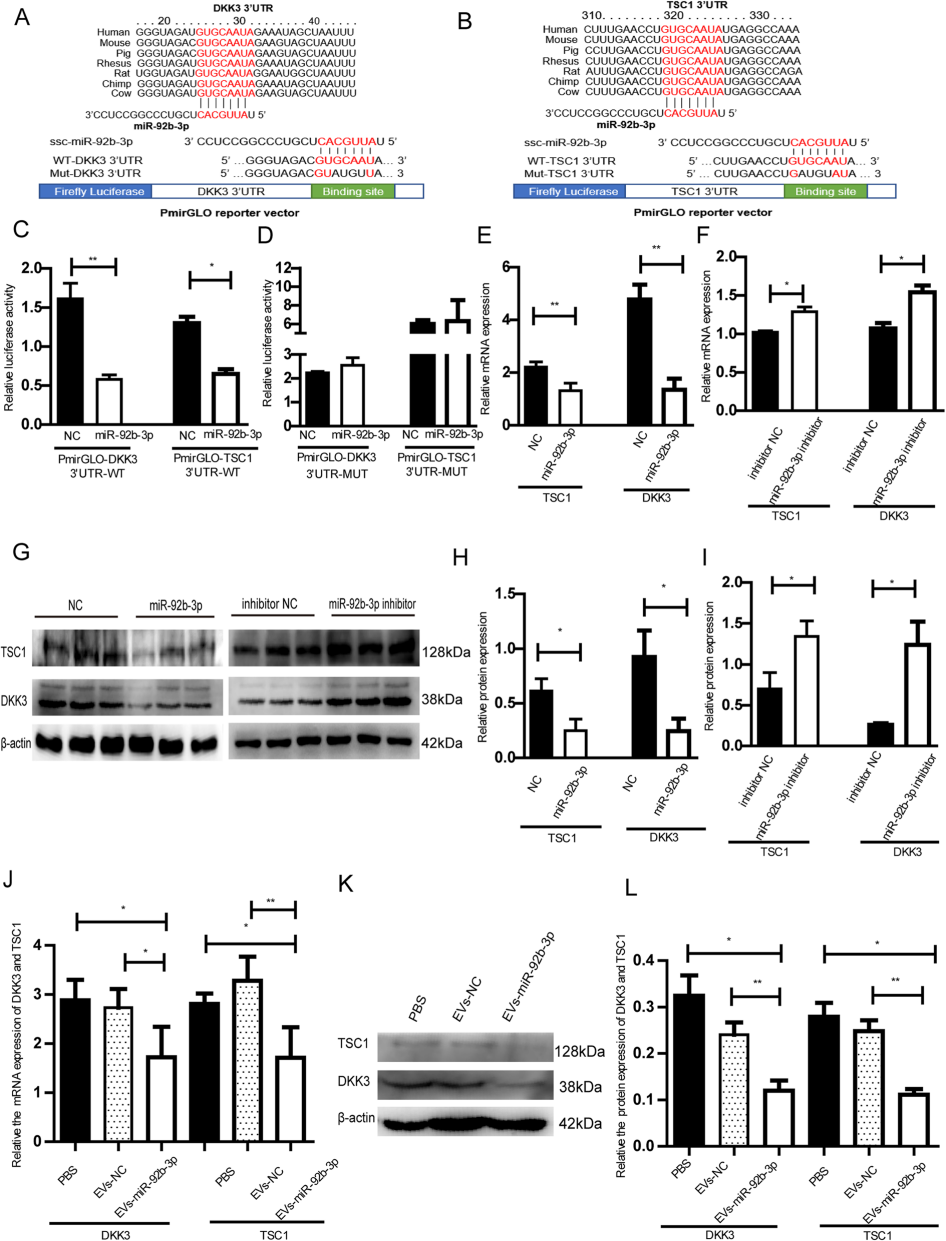
TSC1 and DKK3 are direct targets of miR-92b-3p. **A B** The conservation of the miR-92b-3p binding sites in the 3’UTR of DKK3 and TSC1 from 7 different species, and the predicted binding site and mutated site of miR-92b-3p in the 3’UTR of porcine DKK3 or TSC1. **C D** PTr2 cells were co-transfected with PmirGLO-DKK3-3' UTR (Wt or Mut) or PmirGLO-TSC1-3' UTR (Wt or Mut) and the indicated RNA oligonucleotides (NC and miR-92b-3p). * *P* < 0.05, * *P < 0.01. RT-qPCR (**E**) (**F**) and Western blot (**G**) (**H**) (**I**) analysis of DKK3 and TSC1 after transfection with miR-92b-3p mimic or inhibitor in PTr2 cells. **J** EVs-miR-92b-3p inhibited the mRNA expression level of DKK3 and TSC1; **K L** After the treatment of EVs-miR-92b-3p, the relative protein expression level of TSC1 and DKK3. The β-actin was used as the internal control of Western Blot. Data are mean ± s.d., derived from three independent experiments. * *P* < 0.05, * *P < 0.01

To investigate the regulatory mechanism of miR-92b-3p in PTr2 cells, public websites (such as miRDB, miRBase and TargetScan) were used to predict target genes of miR-92b-3p. After our screening, *DKK3* and *TSC1* were selected for further study. The target site sequences in the 3′-UTR of *DKK3* and *TSC1* show high conservation in mammals, including humans, mice and pigs (Fig. 7A, B). We verified whether miR-92b-3p targets the *DKK3* and *TSC1* 3′-UTR by constructing luciferase reporter plasmids carrying the *DKK3* or *TSC1* 3′-UTR with wild-type or base-pair mutant (Mut) binding region (Fig. 7A, B). Luciferase activity was obviously decreased when PK-15 cells were co-transfected with miR-92b-3p mimic and wild-type luciferase reporters (PmirGLO-DKK3-3’UTR-WT or PmirGLO-TSC1-3’UTR-WT) (Fig. 7C). However, there was no significant difference in the luciferase activity of Mut-type reporters (PmirGLO-DKK3-3’UTR-Mut or PmirGLO-TSC1-3’UTR-Mut) (Fig. 7D). Then, we found the down-regulation of DKK3 and TSC1 expression at mRNA and protein levels after PTr2 cells transfected with miR-92b-3p (Fig. 7E, F, G, H, I). Furthermore, after the treatment of EVs-miR-92b-3p the mRNA and protein levels of *TSC1* and *DKK3* were also down-regulated in PTr2 cells (Fig. 7J, K, L).

### PmirGLO-DKK3-3’UTR-WT or PmirGLO-TSC1-3’UTR-WT rescued the influence of miR-92b-3p in PTr2 cells

**Fig. 8:**
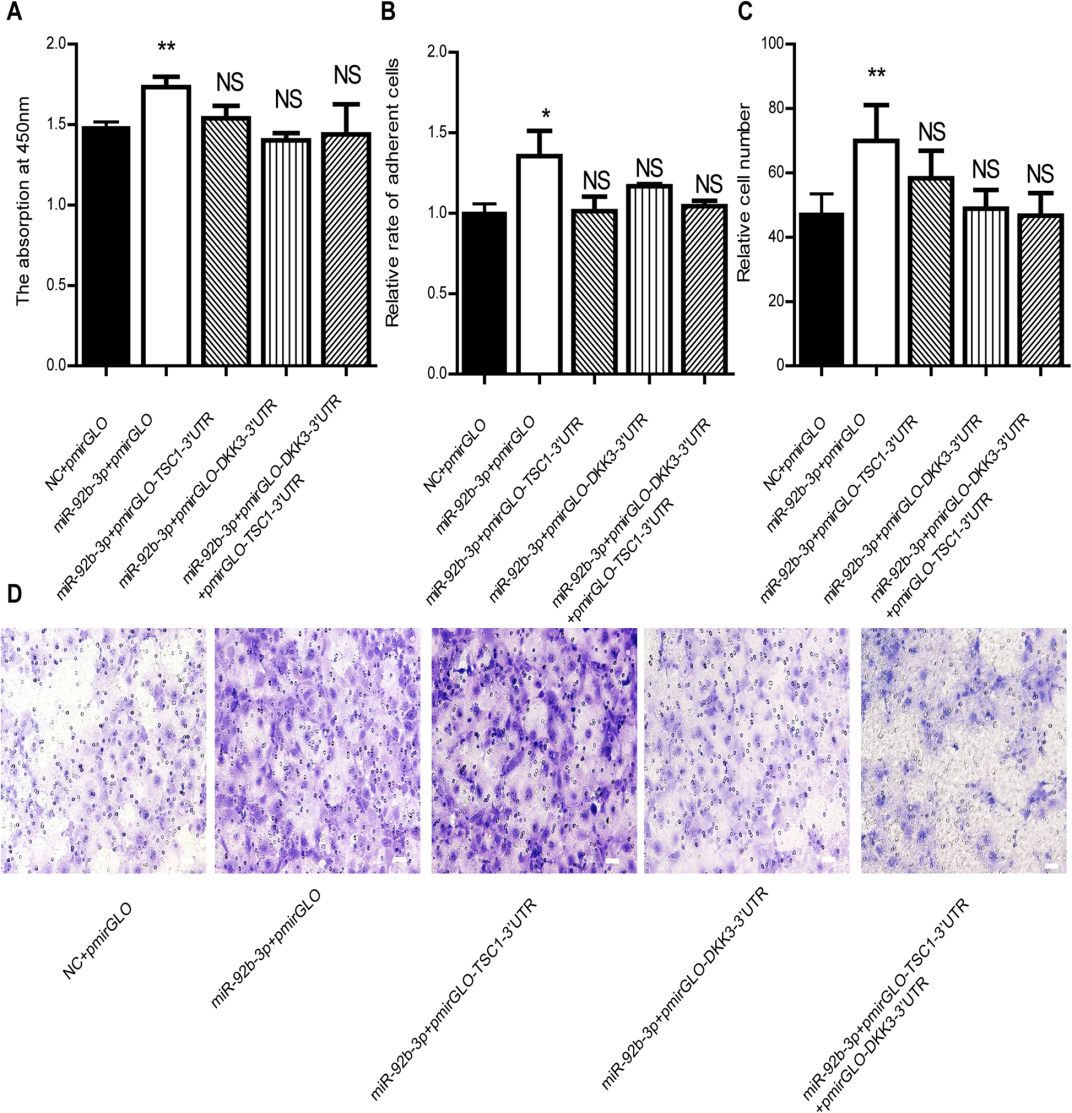
The pmirGLO-TSC1-3’UTR and pmirGLO-DKK3-3’UTR rescue the effect of miR-92b-3p on PTr2 cells. **A B** The pmirGLO-TSC1-3’UTR and pmirGLO-DKK3-3’UTR rescue the effect of miR-92b-3p on proliferation and adhesion in PTr2 cells. **C D** The pmirGLO-TSC1-3’UTR and pmirGLO-DKK3-3’UTR rescue the effect of miR-92b-3p on migration in PTr2 cells. scale = 20 μm. * *P* < 0.05, * *P < 0.01, NS: Not Significance

To verify whether miR-92b-3p regulates the proliferation, adhesion and migration of PTr2 cells through targeting *DKK3* and *TSC1*, PmirGLO-DKK3-3’UTR-WT or PmirGLO-TSC1-3’UTR-WT was transfected to restore the effect of miR-92b-3p on PTr2 cells. Compared with co-transfection with miR-92b-3p and PmirGLO, co-transfection with miR-92b-3p and PmirGLO-DKK3-3’UTR-WT or PmirGLO-TSC1-3’UTR-WT significantly hindered the proliferation, adhesion and migration of EECs (Fig. 8A, B, C, D). These results indicated that 3’UTR plasmids of *DKK3* or *TSC1* could alleviate the effects of miR-92b-3p on the proliferation, adhesion and migration of PTr2 cells.

## Discussion

Zhou et al. observed the expression levels of miR-92b-3p is up-regulated in the embryo implantation group (day 9, 12 and 15 of pregnancy) compared with the non-pregnant group (day 0, 9, 12 and 15 of estrus) [31], and our previous research suggested that the expression level of miR-92b-3p was higher in EVs from uterine flushing fluids on day 13 of pregnancy than them on day 10 and day 18 of pregnancy [20]. In the current study, we aimed to elucidate the role of miR-92b-3p in EVs during the embryo implantation period. We found that the EVs-miR-92b-3p could be taken up by PTr2 cells, and significantly promoted the proliferation, migration and adhesion of these cells. To further delineate the regulatory mechanism of miR-92b-3p, our study showed that miR-92b-3p plays a vital role in PTr2 cells via targeting *TSC1 and DKK3.*

EVs derived from the endometrium could mediate embryo-maternal communication to promote embryo implantation [32]. In this study, we extracted EVs from the culture medium of porcine primary EECs and identified the EVs derived from EECs as 80-190 nm cup-shaped vesicles positive for TSG101, GAPDH and HSP70 but negative for Calnexin by Western blotting, TEM and NTA. Greening et al. found that the fluorescence-labeled EVs released by human endometrial cancer cells (EEC1) can be absorbed by HTR8 trophoblast cells [33]. In our study, the fluorescence-labeled EVs derived from EECs could be taken up by PTr2 cells. In order to determine that miR-92b-3p can be transferred from EECs to PTr2 cells under the encapsulation of EVs. It has been proved that macrophages can secrete miRNA-containing EVs, which are then efficiently transported into 3T3-L1 adipocytes through the Transwell co-culture system [34]. This study shows that the fluorescent FAM-miR-92b-3p can be secreted from transfected EECs and then be absorbed by PTr2 cells through the Transwell co-culture system. Then we treated PTr2 cells with EVs derived from EECs transfected with miR-92b-3p, and found that the expression of miR-92-3p on PTr2 cells was significantly up-regulated. This experiment further confirmed that miR-92b-3p could be transferred from EECs to PTr2 cells under the encapsulation of EVs.

Successful embryo implantation and placental development require the ability of trophoblast cells to proliferate, migrate and adhere [35, 36]. After PTr2 cells were treated with EVs-miR-92b-3p derived from EECs, it was found that EVs-miR-92b-3p could significantly enhance the proliferation, migration and adhesion of PTr2 cells than EVs-NC and PBS. It is worth noting that EVs-NC derived from EECs can significantly elevate the adhesion of PTr2 cells compared with PBS. Previous studies have shown that EVs derived from EEC1 (human endometrial cancer cell) can promote HTR8 trophoblast adhesion through the FAK signaling pathway [33]. It can be inferred that the EVs of EECs could promote the adhesion of PTr2 cells, and the EVs-miR-92b-3p derived from EECs can further improve the adhesion ability of PTr2 cells. We also found that miR-92b-3p mimic could significantly promote the proliferation, migration and adhesion of PTr2 cells, while miR-92b-3p inhibitor could significantly inhibit the proliferation, migration and adhesion of these cells. Overall, these data suggested that EVs-miR-92b-3p derived from EECs could affect the proliferation, migration and adhesion of PTr2 cells.

The miRNAs could regulate the function of the trophoblast cells to affect the process of embryo implantation by regulating target genes [37]. *TSC1* has been identified as a target gene of miR-92b-3p in Caki-2 cells, vascular smooth muscle cells and follicles [38–40]. In our study, we proved that miR-92b-3p can down-regulated *TSC1* at the mRNA and protein levels in PTr2 cells. In addition, we first proved that DKK3 is one of the target genes of miR-92b-3p. The protein expressions of *TSC1* and *DKK3* were significantly down-regulated in PTr2 cells after the treatment of EVs-miR-92b-3p derived from EECs. Previous studies have shown that *TSC1* knockout can affect mouse embryonic development and affect endometrial receptivity by activating the mTOR pathway [41]. *DKK3* may affect the process of embryo implantation by encoding proteins secreted by human trophoblast cells and embryonic stem cells [42]. Accordingly, the 3'UTR vectors of *TSC1* and *DKK3* (PmirGLO-TSC1-3'UTR and pmirGLO-DKK3-3'UTR) can rescue the effect of miR-92b-3p on PTr2 cells. Therefore, this study suggested that EVs-miR-92b-3p could affect the proliferation, migration and adhesion of PTr2 cells by targeting *TSC1* and *DKK3* genes.

In summary, we revealed that EVs-miR-92b-3p derived from EECs could be taken up by PTr2 cells. Moreover, the EVs-miR-92b-3p could regulate the proliferation, migration and adhesion of PTr2 cells by targeting *TSC1* and *DKK3*. We further inferred that EVs-miR-92b-3p from uterine flushing fluids might be involved in the embryo-maternal communication during the embryo implantation period.

## Supplementary Information

**Additional file 1.**

**Additional file 2:** **Table S1.** Primers for gene PCR. **Table S2.** Synthetic oligo sequences. **Table S3.** PCR primers for vector construction. **Figure S1.** The effect of miR-92b-3p on the expression of genes related to embryo implantation in PTr2 cells.

## Data Availability

All data generated during this study are included in this published article.

## Abbreviations

EVs: Extracellular vesicles
EECs: Endometrial epithelial cells
PTr2 cells: Porcine trophoblast cells
TSC1: Tuberous sclerosis complex subunit
DKK3: Dickkopf 3
