# Supplementary material for: Extracellular vesicles derived from endometrial epithelial cells deliver exogenous miR-92b-3p to affect the function of embryonic trophoblast cells via targeting TSC1 and DKK3
Source: Reprod Biol Endocrinol. 2022 Oct 25;20:152. doi: 10.1186/s12958-022-01023-z (PMC9594956; doi:10.1186/s12958-022-01023-z)
Supplement: Supplementary file 2 — Additional file 2: Table S1. Primers for gene PCR. Table S2. Synthetic oligo sequences. Table S3. PCR primers for vector construction. Figure S1. The effect of miR-92b-3p on the expression of genes related to embryo implantation in PTr2 cells. [file 12958_2022_1023_MOESM2_ESM.pdf]

Table S1 Primers for gene PCR

Primer name	Primer sequences(5'-3')
Sus-DKK3-F	CGTCGATGGGAGATGACGAA
Sus-DKK3-R	TGGTTGTCACAGATGGTCCC
Sus-TSC1-F	CCGCGAAGGTGGAAGAGATT
Sus-TSC1-R	ACAACGTCAGCCGAGAAGAG
Sus-PCNA-F	ATGCCTTCTGGTGAATTTGC
Sus-PCNA-R	TTTCCGAGTTCTCCACTTGC
Sus-U6-F	GCTTCGGCAGCACATATACT
Sus-U6-R	TTCACGAATTTGCGTGTCAT
Sus-RPS20-F	CGCTCCTGGCTCACCGCTGTT
Sus-RPS20-R	TGCGGCTGGTGGTGAGGGTGATCC
Sus-MUC1-F	GGGCTTCTGGGACTCTTT
Sus-MUC1-R	AGGTTATAGGTGCCTGCTT
Sus-VEGF-F	CCTTGCTGCTCTACCTC
Sus-VEGF-R	CCAGACCTTCGTCGTT
Sus-cMYC-F	AGAGATGCCATGTGTCCACG
Sus-cMYC-R	ATTGTGTGTCCGCCTCTTGT
Sus-OPN-F	GCTGCTTTCCAACAAAT
Sus-OPN-R	GTCGTCCACATCGTCTG
Sus-IL1β-F	CATAACAATCTTGGGAGGAC
Sus-IL1β-R	AAAGGTAAGCAGGTGGAA
Sus-LIFR-F	GAGCTTTAACATCTGCTGAACTTCT
Sus-LIFR-R	GCTGTAATGCCCCTCCTACA

F:forward primers, R: reverse primers

Table S2 Synthetic oligo sequences

name	sequences(5’-3’)
miR-92b-3p mimic	UAUUGCACUCGUCCCGGCCUCC<br>AGGCCGGGACAGUGCAAUAUU
NC	UUCUCCGAACGUGUCACGUTT<br>ACGUGACACGUUCGGAGAATT
miR-92b-3p inhibitor/antagomir	GGAGGCCGGGACGAGUCAAUA
inhibitor NC	CAGUACUUUUGUGUAGUACAA

Table S3 PCR primers for vector construction

Primer name	Primer sequences(5'-3')
Sus DKK3-WT-F	GGGTTTAAACCAGGCCTGTTCCGTGGGTAG
Sus DKK3-WT-R	TGCTCTAGAGCCCCTCGTGTTGTTTCAAG
Sus TSC1-WT-F	GGGTTTAAACTCCCCTCCCCCTAAATGGTT
Sus TSC1-WT-R	TGCTCTAGACGCTACCAAATAGCTGGGCT
Sus DKK3-MUT-F	TTTCTTTTCTCCCCTCCCCCTAAATGGTTGCGCCC CTTGAACCTGATGTATATGAGGCCAAATTCATTC TCTGAGTCTGACACACCGCGCT
Sus DKK3-MUT-R	AGCGCGGTGTGTCAGACTCAGAGAATGAATTTG GCCTCATATACATCAGGTTCAAGGGGCGCAACC ATTTAGGGGGAGGGGAGAAAAGAAA
Sus TSC1-MUT-F	TTTCTTTTCTCCCCTCCCCCTAAATGGTTGCGCCC CTTGAACCTGATGTATATGAGGCCAAATTCATTC TCTGAGTCTGACACACCGCGCT
Sus TSC1-MUT-R	AGCGCGGTGTGTCAGACTCAGAGAATGAATTTG GCCTCATATACATCAGGTTCAAGGGGCGCAACC ATTTAGGGGGAGGGGAGAAAAGAAA

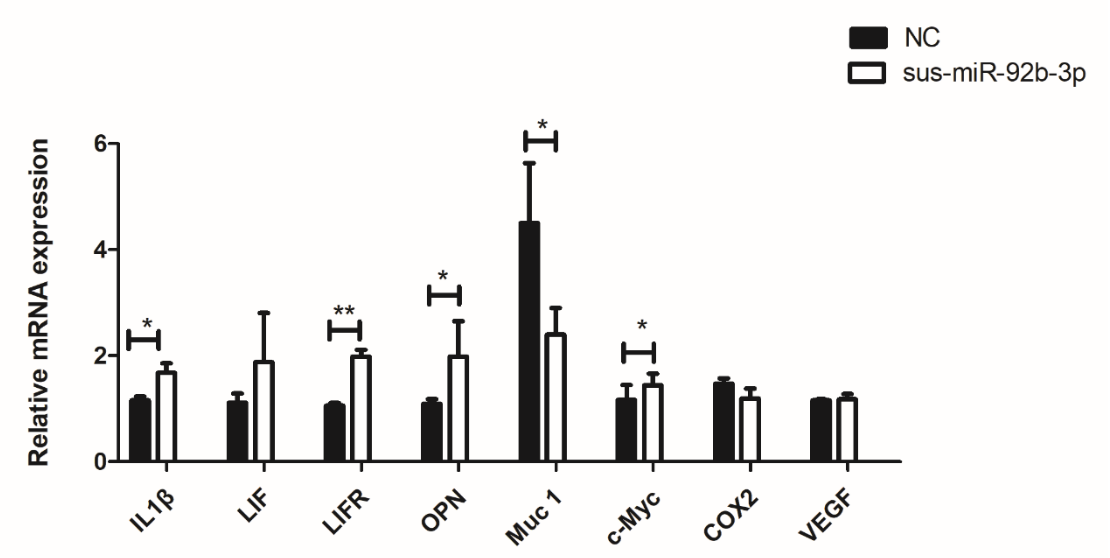

Figure S1 The effect of miR-92b-3p on the expression of genes related to embryo implantation in PTr2 cells.
